# Targeting of RRM2 suppresses DNA damage response and activates apoptosis in atypical teratoid rhabdoid tumor

**DOI:** 10.1186/s13046-023-02911-x

**Published:** 2023-12-20

**Authors:** Le Hien Giang, Kuo-Sheng Wu, Wei-Chung Lee, Shing-Shung Chu, Anh Duy Do, Chun A. Changou, Huy Minh Tran, Tsung-Han Hsieh, Hsin-Hung Chen, Chia-Ling Hsieh, Shian-Ying Sung, Alice L. Yu, Yun Yen, Tai-Tong Wong, Che-Chang Chang

**Affiliations:** 1 International Ph.D. Program for Translational Science, College of Medical Science and Technology, Taipei Medical University, Taipei, 11031 Taiwan; 2 Department of Biology and Genetics, Hai Phong University of Medicine and Pharmacy, Hai Phong, 180000 Vietnam; 3 Graduate Institute of Clinical Medicine, College of Medicine, Taipei Medical University, Taipei, 110 Taiwan; 4 The Ph.D. Program for Translational Medicine, College of Medical Science and Technology, Taipei Medical University, Taipei, 110 Taiwan; 5 Department of Physiology, Pathophysiology and Immunology, Pham Ngoc Thach University of Medicine, Ho Chi Minh City, 700000 Vietnam; 6 The Ph.D. Program for Cancer Biology and Drug Discovery, College of Medical Science and Technology, Taipei Medical University, Taipei, 11031 Taiwan; 7 Department of Neurosurgery, Faculty of Medicine, University of Medicine and Pharmacy, Ho Chi Minh City, 700000 Vietnam; 8 Joint Biobank, Office of Human Research, Taipei Medical University, Taipei, 110 Taiwan; 9 Division of Pediatric Neurosurgery, Neurological Institute, Taipei Veterans General Hospital, Taipei, 112 Taiwan; 10 Laboratory of Translational Medicine, Development Center for Biotechnology, Taipei, 115 Taiwan; 11 Institute of Stem Cell and Translational Cancer Research, Chang Gung Memorial Hospital at Linkou and Chang Gung University, Taoyuan, 333 Taiwan; 12 Genomics Research Center, Academia Sinica, Taipei, 115 Taiwan; 13 Pediatric Brain Tumor Program, Taipei Cancer Center, Taipei Medical University, Taipei, 110 Taiwan; 14 Division of Pediatric Neurosurgery, Department of Neurosurgery, Taipei Medical University Hospital and Taipei Neuroscience Institute, Taipei Medical University, Taipei, 110 Taiwan; 15 Neuroscience Research Center, Taipei Medical University Hospital, Taipei, 110 Taiwan; 16 TMU Research Center for Cancer Translational Medicine, Taipei Medical University, Taipei, 110 Taiwan; 17 The Ph.D. Program for Translational Medicine, College of Medical Science and Technology, Taipei Medical University, 6F., Education & Research Building, Shuang-Ho Campus, No. 301, Yuantong Rd., Zhonghe Dist., New Taipei City, 23564 Taiwan

## Abstract

**Background:**

Atypical teratoid rhabdoid tumors (ATRT) is a rare but aggressive malignancy in the central nervous system, predominantly occurring in early childhood. Despite aggressive treatment, the prognosis of ATRT patients remains poor. RRM2, a subunit of ribonucleotide reductase, has been reported as a biomarker for aggressiveness and poor prognostic conditions in several cancers. However, little is known about the role of RRM2 in ATRT. Uncovering the role of RRM2 in ATRT will further promote the development of feasible strategies and effective drugs to treat ATRT.

**Methods:**

Expression of RRM2 was evaluated by molecular profiling analysis and was confirmed by IHC in both ATRT patients and PDX tissues. Follow-up in vitro studies used shRNA knockdown RRM2 in three different ATRT cells to elucidate the oncogenic role of RRM2. The efficacy of COH29, an RRM2 inhibitor, was assessed in vitro and in vivo. Western blot and RNA-sequencing were used to determine the mechanisms of RRM2 transcriptional activation in ATRT.

**Results:**

RRM2 was found to be significantly overexpressed in multiple independent ATRT clinical cohorts through comprehensive bioinformatics and clinical data analysis in this study. The expression level of RRM2 was strongly correlated with poor survival rates in patients. In addition, we employed shRNAs to silence RRM2, which led to significantly decrease in ATRT colony formation, cell proliferation, and migration. In vitro experiments showed that treatment with COH29 resulted in similar but more pronounced inhibitory effect. Therefore, ATRT orthotopic mouse model was utilized to validate this finding, and COH29 treatment showed significant tumor growth suppression and prolong overall survival. Moreover, we provide evidence that COH29 treatment led to genomic instability, suppressed homologous recombinant DNA damage repair, and subsequently induced ATRT cell death through apoptosis in ATRT cells.

**Conclusions:**

Collectively, our study uncovers the oncogenic functions of RRM2 in ATRT cell lines, and highlights the therapeutic potential of targeting RRM2 in ATRT. The promising effect of COH29 on ATRT suggests its potential suitability for clinical trials as a novel therapeutic approach for ATRT.

**Supplementary Information:**

The online version contains supplementary material available at 10.1186/s13046-023-02911-x.

## Introduction

Central nervous system (CNS) atypical teratoid rhabdoid tumor (ATRT) is an aggressive and lethal human pediatric brain cancer. ATRT can arise anywhere in the CNS, but mostly occurs in the cerebellum or brain stem [1–3]. This tumor has been characterized by inactivation of *SMARCB1* or rarely *SMARCA4*, which encodes hSNF5/BAF47/INI1 and BRG1, respectively. Both hSNF5/BAF47/INI1 and BRG1 act as core subunits of ATP-dependent chromatin remodeling SWI/SNF complex [4, 5]. The perturbation of SWI/SNF complexes plays an important role in epigenetic alteration, tumorigenesis, lineage specification, and maintenance of stem cells, especially in the regulation of gene expression programs [6]. Based on (epi)-genomic profiles, ATRT has been defined to three subgroups including ATRT-TYR, ATRT-SHH, and ATRT-MYC [7, 8]. Although ATRT only represents 1–2% of all pediatric CNS tumors, it is the most common malignant brain tumor in children younger than 1 year of age [9]. It has been decades since ATRT was first described, unfortunately, the absence of a standardized treatment still persists. The multimodal approach has become the mainstay of ATRT treatment with safe surgical resection followed by chemotherapy and radiotherapy [9–11]. Recent advancements in our understanding of ATRT biology and development of model therapeutics have led to notable improvement in survival rate of ATRT patients in recent years [5], despite reports indicating a persistently poor prognosis for ATRT [12].

In recent years, the advancement and utilization of bioinformatics in analyzing (epi)-genetics profiles and clinical data has significantly facilitated the identification of numerous novel therapeutic targets. As a result, certain drugs have been subjected to clinical trials [4]. Nevertheless, several adverse effects and high relapse rate of current treatment regimens have underscored the importance and necessity to develop highly efficacious, targeted therapies for this disease. Recent studies have evaluated the promising role of RRM2, a subunit of ribonucleotide reductase (RNR), as a target against brain tumors. Overexpression of RRM2 associated with genesis, progression of neuroblastoma [13], promotes tumorigenesis in glioblastoma [14, 15], glioma [16] and is a potential prognosis biomarker of these tumors [17–19]. RRM2 is among the top 10% of most overexpressed genes in cancer analyses using the ONCOMINE database [20]. Upregulation of RRM2 has been associated with tumor angiogenesis, metastasis, progression, as well as poor prognosis of patients [20–22]. A combination of RRM2 and CHK1 inhibitors has shown synergistic effects in neuroblastoma in vitro and in vivo [23]. Additionally, when examining RNA-sequencing (RNA-seq) data for potential therapeutic targets, Birks et al. demonstrated that RRM2 is prominently up-regulated in all three rhabdoid tumor subsets (comprising two ATRTs and one kidney rhabdoid tumor) compared to normal tissues [24]. Moreover, the protein-protein interaction (PPI) network revealed that RRM2 belongs to 15 hub genes that may serve as diagnostic and therapeutic markers of ATRT [25].

In decades, inhibitors targeting RNR have been investigated as cancer chemotherapeutic agents to treat multiple myeloma, lymphoma, or solid tumors as well [26]. However, RNR inhibitors such as gemcitabine can inhibit numerous off-targets [27] and conferring high rates of therapeutic resistance [28]. Another drug is hydroxyurea, which has limited use due to poor efficacy, inconvenient dosing schedule, and a high rate of resistance in patients [27]. On the other hand, COH29 is a novel inhibitor of RRM2, and it can overcome hydroxyurea- and gemcitabine- resistance in ovarian cancer and leukemia cells [29]. COH29 is an aromatically substituted thiazole compound that occupies a structurally conserved ligand-binding pocket on the RRM2 subunit. Moreover, COH29 is not an iron chelator (as 3-AP), thus reducing the potential side effects [27, 29]. COH29 is currently undergoing phase 1 clinical trial evaluation for the treatment of patients with solid tumors.

In this study, overexpression of RRM2 was found in ATRT samples and associated cell lines. Knockdown of RRM2 significantly decreased cell proliferation, cell migration, and induced apoptosis in ATRT cells. COH29 treatment activated DNA damage, inhibited cell growth and survival in vitro, and suppressed tumor growth and prolonged survival rate in vivo. In light of those potentials, our study suggests that RRM2 may be a novel therapeutic target, and COH29 is a promising drug for the ATRT treatment.

## Materials and methods

### Patient cohorts

This study included 28 patients who had a diagnosis of ATRT from 2007 to 2022 at Taipei Medical University Hospital (TMUH) and Taipei Veterans General Hospital (VGH) and were aged < 18 years at diagnosis. This study was conducted in accordance with the Declaration of Helsinki, and all patients provided written informed consent. Patient information was completely anonymized. The study protocol was approved by the Institutional Review Board of Human Subjects Research Ethics Committee of the Taipei Medical University and Taipei VGH, Taiwan (IRB approval numbers: N201901033 and 2019-02-010C, respectively).

### Cell culture

Human ATRT cell lines (BT12, CHLA266, CHLA02, CHLA04, and Re1P6) were cultured as previously described [30]. HEK293T cell line was cultured in DMEM medium (Thermo Fisher Scientific, MA, USA cat# 12439054) with 10% FBS. All the mediums were added 1% antibiotic-antimycotic (Santa Cruz Biotechnology, CA, USA, cat# sc-3690), and all cell lines were incubated at 37 °C in humidified incubator containing 5% CO_2_. Cells were confirmed to be Mycoplasma-free using the Mycoplasma PCR Detection Kit (abm, Canada).

### Lentiviral transduction and cell transfection

To generate stable gene knockdown cell lines, short hairpin (sh)RNA lentiviral expression system was used. The lentiviral vector pLKO.1-puro carrying shRNA sequences shRRM2#1 (TRCN0000038962 target sequences GCTCAAGAAACGAGGACTGAT), shRRM2#2 (TRCN0000286353 target sequences GCAGACAGACTTATGCTGGAA), and shLuc (target sequence CTTCGAAATGTCCGTTCGGTT). The pLKO.1-shRNA and packaging plasmids pCMV-ΔR8.91, pMD.G were purchased from the National RNAi Core Facility (Academia Sinica, Taiwan). Construct contain short hairpin RNA shRRM2#1, shRRM2#2 or shLuc were produced in HEK293T cells with packaging plasmids pCMV-ΔR8.91 and pMD.G using transfection reagent Polyjet (SignaGen Laboratories, MD, USA, cat# SL100688). The medium supernatant containing the lentivirus was collected, filtered through a 0.45 µm filter, and supplemented with 8 µg/mL polybrene to infected in ATRT cells. Cells were incubated for 24 h at 37 °C, then replaced by complete medium to allowed cells recover for 48 h. Stable knock-down cell lines were selected by puromycin (Gibco, USA, cat# A1113803) for at least 72 h. Knockdown efficiency was assessed by immunoblotting and cells transfected with shLuc were used as control group.

### Cell viability (IC50) assay

COH29 (MedChemExpress, NJ, USA, HY-19931) was dissolved in dimethyl sulfoxide (DMSO) (Sigma-Aldrich, Germany, D2650) then diluted to serial concentration in medium. BT12, CHLA266, and Re1P6 cells were seeded into 96-well plate with 5000 cells per well, 6 replicate wells per plate. Cell growth inhibition assay was assessed with CCK8 kit (Abcam, UK, ab228554) by a microplate reader after 48 h of exposure to a serial dilution of COH29 (0.5–100 µM). The half-maximal inhibitory concentration (IC50) of COH29 in BT12, Re1P6, and CHLA266 cells was analyzed using AT Bioquest tool (https://www.aatbio.com).

### Cell proliferation assay

Cells were seeded into 96-well plates with 1000, 2500 and 2500 cells per well for BT12 - CHLA266 - Re1P6 respectively, 6 replicate wells per plate. Cells were treated with different dilutions of COH29: 4-8-16-20 µM (for BT12) or 3.5-7-14 µM (for Re1P6 and CHLA266), and 0.1% DMSO as control. Cell growth was assessed every 24 h, then 20µL CCK8 reagent was added to each well before measuring the optical density at 460 nm by microplate reader.

### Colony formation assay

Stable knockdown cells or parental cells were seeded into 6-well plates with 2000, 4000 and 8000 cells per well for BT12, CHLA266 and Re1P6, respectively. For drugs treatment, medium with COH29: 2-4-6 µM (for BT12), 3-5-7 µM (for Re1P6), 1-3-5 µM (for CHLA266), or 0.1% DMSO were changed one day after seeding parental cells. Colonies formed after 10 days (for BT12) and 20 days (for CHLA266 and Re1P6) were washed with PBS 1X, fixed with 4% formaldehyde (Macron, cat# H121-08), and stained with 0.5% crystal violet.

### Migration assay

Wound-healing assay and transwell assay were applied for detecting cell migration ability. For the transwell assay, 10^5^ cells in 500µL serum-free medium were seeded into transwell upper chamber (Corning, MA, USA, cat# 353097) and placed in 24-well plates with 500µL medium containing 10% FBS. For the drug treatment experiment, 10^5^ cells in 250µL serum-free medium were seeded in the upper chamber for 4 h before adding 250µL serum-free medium containing 2X concentration of COH29 or 0.2% DMSO. After 16-20-24 h incubation for BT12, CHLA266 and Re1P6 respectively, non-migrated cells in the upper chamber were carefully removed using cotton swab. The migrated cells were fixed with 4% formaldehyde and stained with 0.5% crystal violet. Finally, the samples were observed for recording images of cell migration. Ten random fields of each insert were photographed at 20X magnification and quantified using ImageJ.

Wound-healing assay was proceeded using commercial chambers (ibidi, Germany, cat# 80209). Firstly, chambers were fixed in 6 cm dishes, then 100µL of 2 × 10^4^ cells were seeded in to each well of chamber. After 24 h for cell attachment, the chambers were gently taken out and washed with PBS. For the knockdown experiment, 4 mL of culture medium was added to further cultivating the cell culture. For the drugs treatment experiment, cells were treated with medium containing COH29: 8-16-20 µM (for BT12) or 3.5-7-14 µM (for Re1P6 and CHLA266), or 0.1% DMSO. Images were acquired at 0-24-48-72 h. Finally, the average wound gap between wound edges were calculated for cell movement. Images were obtained from 6 different fields using microscope, wound closure and migration efficiency were analyzed using ImageJ.

### Western blot

Cell lysates were incubated in RIPA buffer plus EDTA-free Protease Inhibitor Cocktail (Roche Applied Science, Germany, cat# 4693132001) for 30 min, then centrifuged at 13000xg for 20 min at 4 °C. The supernatant fluid was collected to measuring protein concentration using BCA Protein Assay Kit (Thermo Fisher Scientific, USA, cat# 23225). Human normal brain tissue lysate was procured from GeneTex, Inc (cat# GTX28771). Equal amounts of samples were loaded for western blot analysis in 4–15% SDS-PAGE gel. Proteins were transferred on a nitrocellulose membrane (0.45 µm NC), blocked with 5% non-fat mill in PBS 1X with 0.1% Tween 20 (PBST) and probed with primary antibodies overnight at 4 °C. The primary antibodies used were: Beta actin (Epitomics, cat# S0861), RRM2 (GeneTex, cat# GTX103193), TOP2A (GeneTex, cat# GTX35137), TTK (Proteintech, cat# 10381-1-AP), PBK (GeneTex, cat# GTX60560), KIF20A (Santa Cruz Biotechnology, cat# SC-374508), TGFB3 (Abclonal, cat# A8460), MUC1 (Proteintech, cat# 19976-1-AP), Caspase 9 (Abclonal, cat# A13682), Cleaved Caspase 9 (Cell Signaling Technology, cat# 95016), Caspase 7 (Cell Signaling Technology, cat# 9492), Cleaved Caspase 7 (Cell Signaling Technology, cat# 9491), Caspase 3 (Cell Signaling Technology, cat# 9662), Cleaved Caspase 3 (Cell Signaling Technology, cat# 9661), Survivin (Abclonal, cat# A1551), MCL1 (Abclonal, cat# A0250), PARP (Cell Signaling Technology, cat# 9532), BAD (Cell Signaling Technology, cat# 9292), BAX (GeneTex, cat# GTX61026), Cleaved PARP (Cell Signaling Technology, cat# 9541), H2AX (GeneTex, cat# GTX108272), H2AX-γ (GeneTex, cat# GTX127340), BRCA1 (Abclonal, cat# A11549), RAD51 (Abclonal, cat# A6268). After washing with PBST, the membranes were incubated with corresponding HRP conjugated Goat anti-Mouse or HRP conjugated Goat anti-Rabbit (Jackson ImmunoResearch Inc). The membranes were washed with PBST (three times for 10 min) before visualized by enhanced chemiluminescence ECL (PerkinElmer, MA, USA).

### Apoptosis assay

Apoptosis assay was performed using an eBioscience Annexin V Apoptosis Detection Kit FITC (Invitrogen, MA, USA, cat# 88800574) based on the manufacturer’s manual. Briefly, cells were collected and washed, followed by centrifugation then re-suspended in 1X binding buffer. Then, 5µL fluorochrome-conjugated Annexin V was added in 100µL of the cell suspension and incubated for 15 min at room temperature. Finally, the cells were washed, centrifuged and re-suspended in 200µL 1X binding buffer before adding 5µL propidium iodide staining solution (PI). The stained cells were analyzed using FACS Canto II cytometer (BD Biosciences, NJ, USA).

### Comet assay

Re1P6 and CHLA266 cells were seeded in 6 cm dishes and incubated overnight at 37 °C. The next day, cells were treated with COH29 (14-25-50 µM) or 0.1% DMSO for 10 h. Cells were collected and the alkaline comet assay was applied according to the manufacturer’s instructions of Comet Assay kit (R&D System, Bio-Techne, MN, USA, cat# 4250-050-K). The individual cells or comets were viewed and photographed using a fluorescent microscope (Zeiss) equipped with an DAPI filter. To evaluate DNA damage, a total of 100 individual cells per sample were used to calculate the tail DNA percentage. The photographs were analyzed using Comet Score 2.0.0.38 software (TriTek Corp.).

### Histological stains and immunohistochemistry

Tissue specimens were embedded in paraffin after fixing in 4% paraformaldehyde. Further, thin sections of tissue embedded in paraffin were cut and stained with hematoxylin and eosin (HE) or used for the immunohistochemistry (IHC) analysis. IHC assay was performed according to the manufacturer’s instructions (Novolink Polymer Detection Systems, Leica, Germany). Briefly, tissue specimens were de-paraffinized in xylene, and rehydrated in serial ethanol solutions. Antigen retrieval was achieved by incubation in antigen retrieval buffer (pH 6.0) in a pressure cooker for 10 min at high pressure. The first antibodies RRM2 (Sigma-Aldrich, cat# HPA056994), Cleaved Caspase 3 (Cell Signaling Technology, cat# 9661) were incubated overnight at 4 °C. Then the sections were incubated in the appropriate secondary antibody and processed with DAB Chromogen before staining with hematoxylin. Normal brain control tissue slides (HuFPT017), and appropriate positive control (colon tissue - HuFPT036) and negative controls (liver tissue - HuFPT074) from US Biomax, Inc (MD, USA) were included in the IHC staining. Images were captured and analyzed using Motic DSAssistance 4 K (Motic).

### RNA-sequencing of cell lines treated with COH29

BT12 and Re1P6 cell were treated with COH29 (16 µM and 14 µM respectively), then total RNA of indicated samples were isolated using the Trizol Reagent (Invitrogen, MA, USA) and quantified in a NanoDrop (ThermoFisher Scientific, USA). The samples were sent to the Biotools Microbiome Research Center (Taiwan) for library preparation and sequencing.

### Orthotopic ATRT xenograft model

The orthotopic ATRT xenograft model was performed according to previously described procedures [30]. This research has been approved by TMU Ethic Committee for ATRT orthotopic xenograft mice model (LAC-2020-0219 and LAC-2021-0517). Tumor formation confirmed by MRI was performed at day 20 after injection (pre-treatment). From day 21, the mice with tumor formation confirmed by MRI were randomly divided into control group (treated with Kolliphor HS15 in saline; *n* = 8) and COH29 treatment group (treated with COH29 (400 mg/kg) by oral gavage every day in 3 weeks; *n* = 8). The post-treatment brain MRI was obtained at day 28. Mice were monitored the irreversible neurological deficits and body weight daily. Tumor volumes were calculated based on post-contrast T1-weighted sequences using Image J. All animal care and experimental studies were performed according to the guidelines and approval of the TMU Animal Center.

### Gene expression profiles and clinical data analysis

Data for clinical cohorts published by other studies were analyzed on R2 Genomics Analysis and Visualization Platform (https://hgserver1.amc.nl), and PedcBioPortal for Integrated Childhood Cancer Genomic (https://pedcbioportal.org). Genetic dependencies and gene expression of cell lines were analyzed by Depmap Portal tool (https://depmap.org). ATRT patients data and ATRT-PDX data have been described in our previous study [30]. RNA-seq and clinical data were analyzed in Biotools RNA-seq v1.6.4 and in R environment. Differentially expressed genes threshold (FDR < 0.05) was used as a cutoff for differential expression assessment. The RNA-Seq data of the 28 patients are available in the Gene Expression Omnibus (GSE218948).

### Statistical analysis

All experiments were performed in triplicate and analyzed using GraphPad Prism version 8.0.1 software. Data were expressed as mean ± standard deviation (SD) or standard error of the mean (SEM), and a *p* value ≤ 0.05 was considered to be statistically significant. Statistical parameters were described in the figures and figure legends.

## Results

### RRM2 functions as an oncogene in ATRT

Due to the highly malignant nature of ATRT, it is imperative to discover and assess new treatment approaches in order to address its aggressiveness effectively. The RNA-seq data from ATRT patient tissues (*n* = 28), ATRT-PDX tissues (*n* = 19) [30], and four normal brain tissues (GSM2501173, GSM2501174, GSM2501175, and GSM2193194) were gathered to evaluate the druggable genes as potential therapeutic targets for ATRT. Eight candidate drug-targetable genes with high expression levels in ATRT were found including FGFR1, TOP2A, KIF20A, PBK, RRM2, TTK, TGFB3, and MUC1 (Fig. S1A and B). Protein expression of these target genes were checked with five ATRT cell lines and human normal brain tissue lysates by immunoblot experiment (Fig. S1C).

**Fig. 1:**
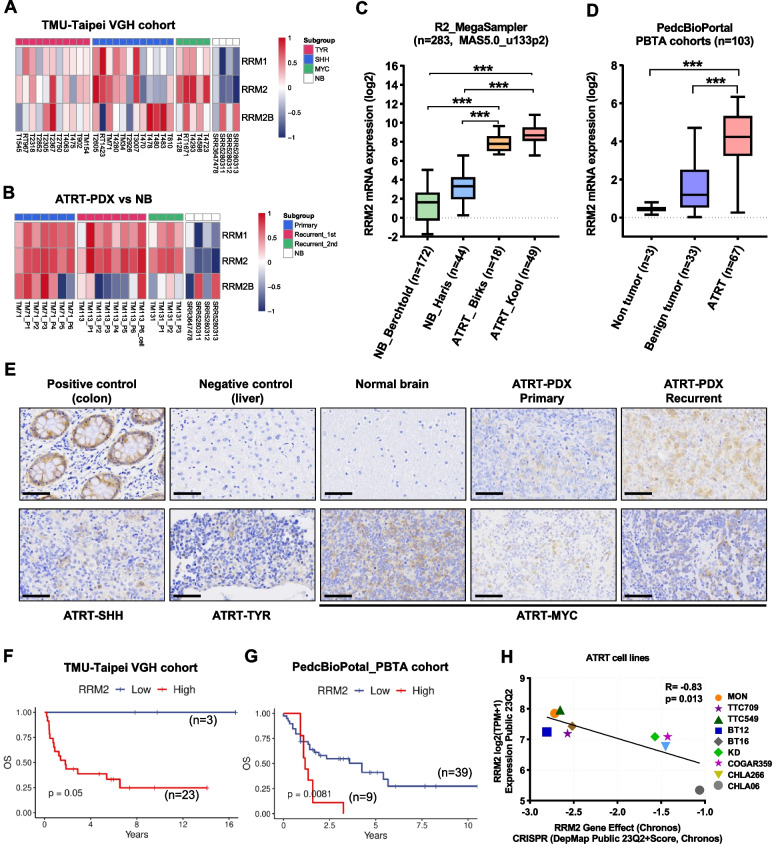
RRM2 expression and its correlation with overall survival in ATRT. **A**, **B** Heatmaps of RRM2-related gene expression in human ATRT (**A**) and ATRT-PDX (**B**) samples compared with normal brain tissues. For normal samples, raw RNA-seq data of four normal brain tissues were downloaded from the GEO data set. **C** Expression levels of RRM2 mRNA in human ATRT and normal brain tissues in public data sets using the R2 Platform. **D** Expression levels of RRM2 mRNA in human ATRT, normal brain, and benign tumor tissues in PedcBioPortal dataset. Bar indicates the mean mRNA levels of each group, data are presented as min to max, Tukey’s multiple comparisons test, ***p < 0.001. RNA expression was normalized using logarithm base 2. **E** Comparison of IHC staining for RRM2 in the colon (as positive control), liver (as negative control), normal brain, human ATRT, and ATRT-PDX samples. Scale bar 30 µm. **F**, **G** The correlation of RRM2 mRNA level with patient’s overall survival (OS) in TMU-Taipei VGH cohort (*n* = 26) (**F**), and PBTA cohort from PedcBioPortal (*n* = 48) (**G**). **H** The correlation between RRM2 gene effect and RRM2 gene expression in nine ATRT cell lines was analyzed from DepMap project. Gene effect means the necessity of RRM2 for the survival of ATRT cell lines was retrieved and analyzed from CRISPR (DepMap Public 23Q2 + Score, Chronos) dataset. A lower score means that a gene is more likely to be dependent in a given cell line, meaning that gene has a higher contribution to cell survival. The RRM2 expression was analyzed from the project Expression Public 23Q2 dataset. Gene expression TPM values of the protein-coding genes for DepMap cell lines. Values are inferred from RNA-seq data using the RSEM tool and are reported after log2 transformation, using a pseudo-count of 1; log2(TPM + 1)

RRM2 has been previously reported as a master driver of tumor aggressiveness and a biomarker of poor prognosis in several cancer including glioma, oral cancer, lung cancer, colorectal cancer, prostate cancer, endometrial cancer and cervical cancer [18, 31–36]. However, the function of RRM2 in ATRT is still unclear. Thus, we clarify the roles of RRM2 in ATRT and exam the therapeutic potential which may further promote the development of feasible and effective drugs or strategies to treat this disease. We analyzed the RNA expression of RNR subunits in our ATRT cohort compared with normal brain tissues. Among the RNR members, RRM2 showed more significant activation than RRM1 and RRM2B (Fig. 1A and B). Interestingly, the similar results were observed in ATRT patients of R2_MegaSampler and PedcBioPortal_PBTA cohorts (Fig. 1C, D, S1D and E). In IHC staining analysis, the RRM2 protein expression level was stronger in ATRT compared with normal brain tissue (Fig. 1E). To determine the correlation of RRM2 level for ATRT survival, we analyzed gene expression with clinical data in TMU-Taipei VGH cohort and PedcBioPortal_PBTA cohort. The results revealed that ATRT patients with high RRM2 expression had shorter survival time than those with low expression (Fig. 1F and G). Furthermore, to confirm the importance of RRM2 for ATRT survival, we analyzed the correlation between RRM2 dependency scores and RRM2 expression level in nine ATRT cell lines using DepMap database. The result revealed that RRM2 is not only highly expressed in ATRT cells (RRM2 log2 (TPM + 1) > 5) but also necessary for ATRT cells survival (dependency scores less than -1). Besides, the Pearson coefficient showed strong linear relationship between these two variables with the higher level of RRM2, the lower value of dependency scores (*R* = -0.83, *p* = 0.013) (Fig. 1H). Together, our data indicate that RRM2 is highly activated and plays an important role in ATRT.

**Fig. 2:**
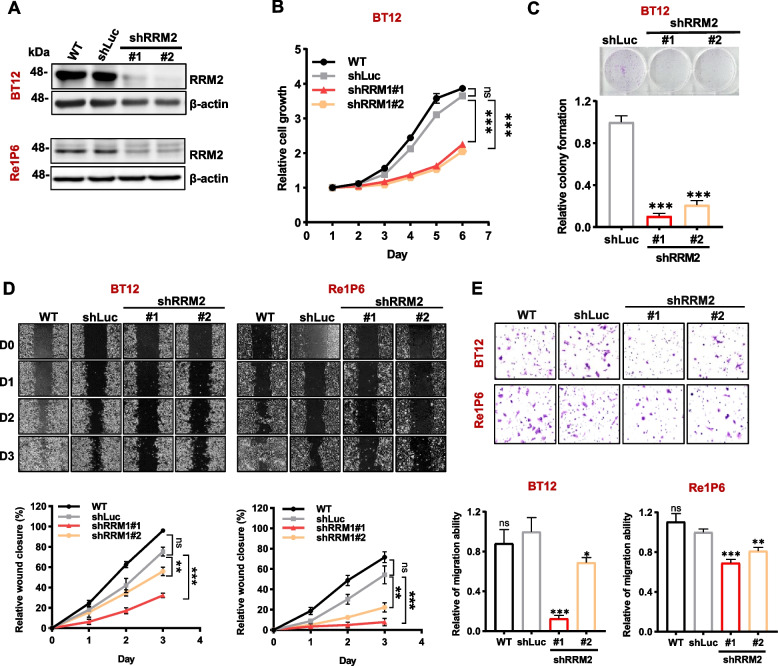
Knockdown of RRM2 suppressed cell growth and decreases the migration capability of ATRT cells. **A** Immunoblotting analyzed RRM2 knockdown efficiency. Endogenous RRM2 expression was inhibited by two independent shRRM2 (shRRM2#1 and shRRM2#2). The parental cells (WT) and luciferase shRNA (shLuc) cells were used as controls. **B**, **C** Knockdown RRM2 attenuates cell growth (**B**) and colony formation abilities (**C**) in BT12 cell. **D**, **E** Wound healing assay (**D**) and transwell assay (**E**) shows the efficiency of RRM2 depletion in ATRT cells. For each experiment, the relative values of parental cells and knockdown cells were compared with the shLuc control. Data are presented as the mean ± SD of triplicated independent experiments, ns non-significant, *p ≤ 0.05, **p ≤ 0.01, ***p ≤ 0.001, Student’s t test

To examine whether RRM2 has oncogenic role in ATRT, we evaluated the functions of RRM2 in ATRT cells. Three RRM2-high expression cells include BT12, CHLA266 cell line, and Re1P6 primary cell were chosen for further experiments (Fig. S1C). Two independent shRNAs (shRRM2#1 and shRRM2#2) were used to inhibit RRM2 expression, and the efficiency of knockdown was evaluated by Western blot assay (Fig. 2A and S2A). Our results demonstrated that the depletion of RRM2 significantly attenuated cell proliferation (Fig. 2B and S2B), colony formation ability (Fig. 2C) of ATRT cells. As cell migration is an important process of cancer development, we performed the wound healing and transwell assay to test the role of RRM2 in this pro-metastatic phenotype. The knockdown of RRM2 suppressed migration ability of both BT12 and Re1P6 cells (Fig. 2D and E). These results proposed that RRM2 has oncogenic properties in ATRT, which suggests that RRM2 may be a promising therapeutic target for this cancer.

### COH29 inhibited ATRT cells growth and migration in vitro

**Fig. 3:**
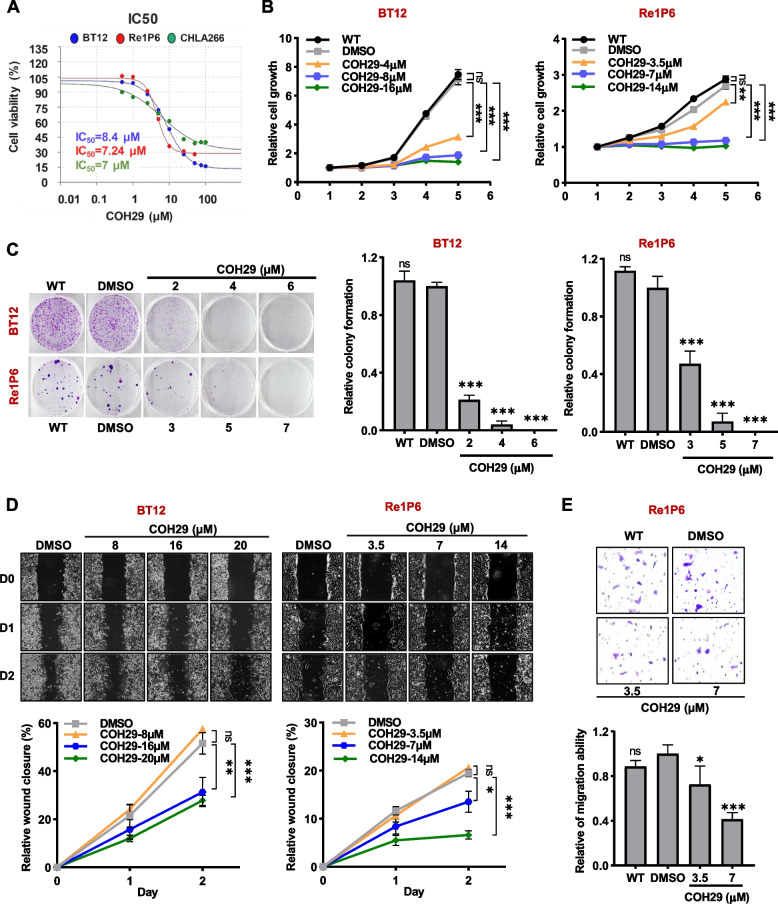
COH29 showed an anticancer effect on ATRT cells. **A** IC50 values of COH29 in BT12, Re1P6 and CHLA266 cells. **B**, **C** Analysis of cell proliferation (**B**) and colony formation (**C**) in COH29-treated cells. **D** Wound healing assay after cells were treated with COH29 in three days. **E** Transwell assay analyzed the migration ability of Re1P6 cells after being treated with COH29. A relative rate of cell migration was analyzed from the number of the cells migrating per chamber compare with the control. In all experiments, 0.1% DMSO was treated as the control. Data are presented as the mean ± SD of triplicated independent experiments, ns non-significant, *p ≤ 0.05, **p ≤ 0.01, ***p ≤ 0.001, Student’s t-test

To further assess the efficacy and specificity of targeting RRM2 in ATRT, we evaluated the pharmacologic effects of COH29, an RRM2 inhibitor on ATRT cells. Firstly, the half-maximal inhibitory concentration (IC50) of COH29 in BT12, Re1P6, CHLA266 cell lines were measured. As shown in Fig. 3A, three ATRT cell lines were highly sensitive to COH29 with an IC50 less than 10 µM. Then, we used different concentrations of COH29 for cell activities analysis. In agreement with the knockdown studies, inhibition of RRM2 activities by COH29 treatment suppressed ATRT cell proliferation (Fig. 3B and S3A), clonogenic capacity (Fig. 3C and S3B), and cell migration ability (Fig. 3D, E, S3C and D).

### COH29 suppressed ATRT tumor growth and prolonged survival rate in vivo

**Fig. 4:**
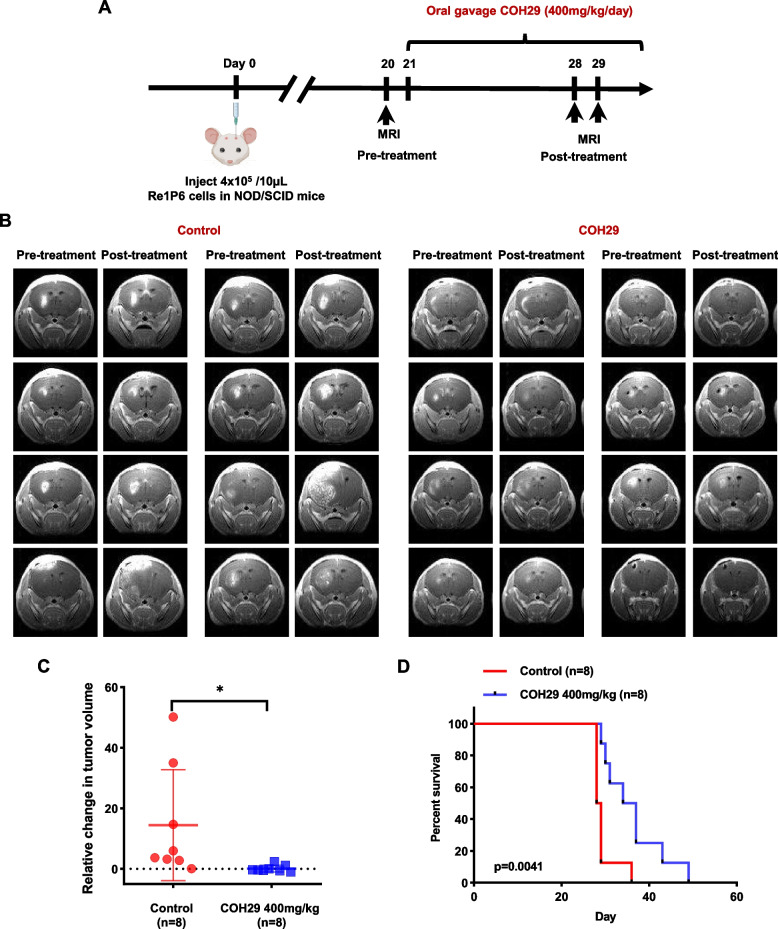
COH29 suppressed tumor growth and prolonged survival of ATRT mice in vivo. **A** In vivo experimental schema. **B** Brain MRI images of mice in the control group and COH29-treated group in pre- and post-treatment. **C** Representative of the change in tumor volume between the COH29-treated group and control group. *p < 0.05, Unpaired t-test. The data are presented as the median ± interquartile range. **D** The overall survival of ATRT mice in control group and COH29-treated group. **p < 0.01, Log-rank (Mantel-Cox) test

On the basis of the anticancer effect in vitro, we hypothesized that COH29 might have therapeutic benefits in mice bearing ATRT. To test this hypothesis, ATRT orthotopic mouse model was utilized (Fig. 4A). MRI images revealed that COH29-treated group had smaller tumors size compared with the control group (Fig. 4B). These results were confirmed by tumor growth analysis. The relative change in tumor volume after COH29 treatment was significantly smaller than those in the control group (Fig. 4C). These observations were further established in terms of mice’s overall survival. Although we noted no major difference in weight loss between the two groups (Fig. S4A), COH29 treatment produced a significant improvement in survival compared with the control group (Fig. 4D). Overall, our data demonstrated that COH29 has an antitumor effect in both in vitro and in vivo model.

### RRM2 inhibition induced apoptosis process in ATRT

**Fig. 5:**
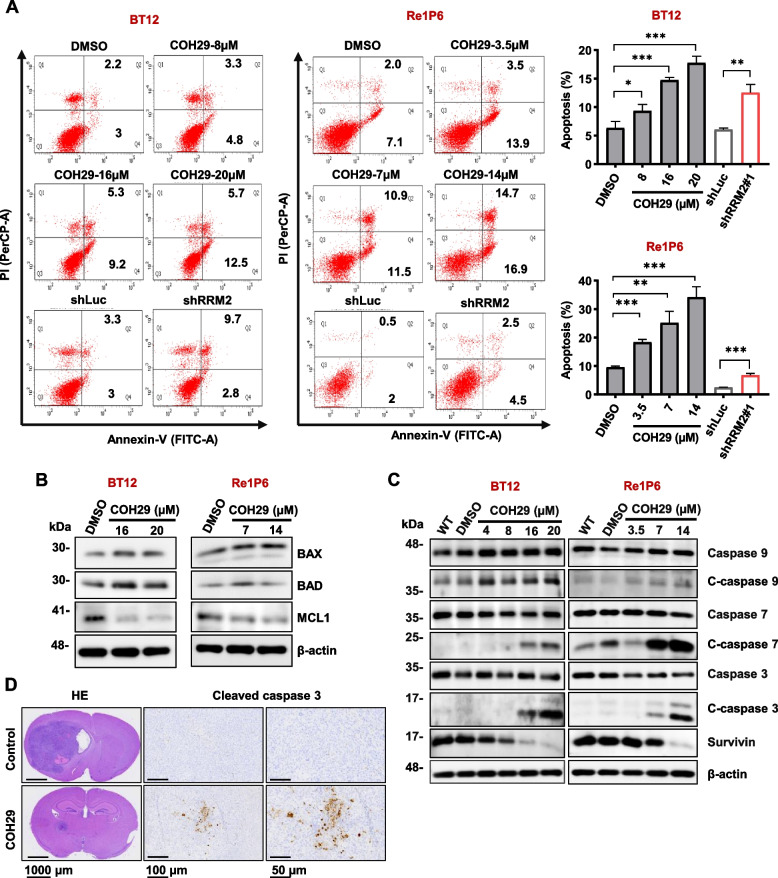
RRM2 inhibition induced apoptosis in ATRT cells. **A** Flow cytometry analysis with Annexin V-PI staining was performed to evaluate the percentage of apoptotic cells. Representative percentage of total apoptotic cells were normalized with control. Data are presented as the means ± SD of three independent experiments. * *p* ≤ 0.05, ** *p* ≤ 0.01, *** *p* ≤ 0.001, Student’s t-test. **B**, **C** Immunoblotting for apoptosis markers of BT12 and Re1P6 cells treated with COH29 in 72 h and 48 h, respectively. **D** HE staining show the size and position of the tumor in the mouse brain. IHC staining detects the expression of cleaved caspase 3 in the ATRT xenograft brain tumor. Scale bar, from left panels to right panels: 1000 µm-100 µm-50 µm

To clarify whether RRM2 inhibition could induce ATRT cells death via apoptosis pathway, ATRT cells were applied for flow cytometry Annexin V-PI staining to analyze their apoptotic states. Results showed that the percentage of apoptotic cells was clearly increased after the inhibition of RRM2 by shRRM2 or COH29 treatment (Fig. 5A and S5A). We examined the protein expression of apoptosis-associated genes with immunoblotting (Fig. 5B, C and S5B). Treatment with COH29 significantly suppressed the expression of anti-apoptotic gene including Survivin and MCL1, and activated the expression of pro-apoptotic genes such as BAX and BAD (Fig. 5B, C and S5B). These results indicated that RRM2 inhibition induced the cell apoptosis in ATRT. Since apoptosis pathway is divided into caspase-dependent and caspase-independent, we next differentiated which apoptosis pathway that COH29 is involved. The factors among the caspase-dependent apoptosis pathway such as cleaved caspase 9, cleaved caspase 7, and cleaved caspase 3 were increased in COH29-treated cells (Fig. 5C and S5B). Notably, cleaved caspase 3 started to increase after 24 h (Re1P6 and CHLA266) or 48 h (BT12), meaning that COH29 could induce apoptosis after a short period of treatment (Fig. S5C). Besides, the signal of cleaved caspase 3 in the tumor specimens was stronger in COH29-treated mice compared with the specimens of control mice (Fig. 5D). Taken together, our data elucidated that RRM2 plays an important role in ATRT survival and COH29 acts as an anticancer agent via activating apoptosis in ATRT in vitro and in vivo.

### COH29 activated DNA damage in ATRT

**Fig. 6:**
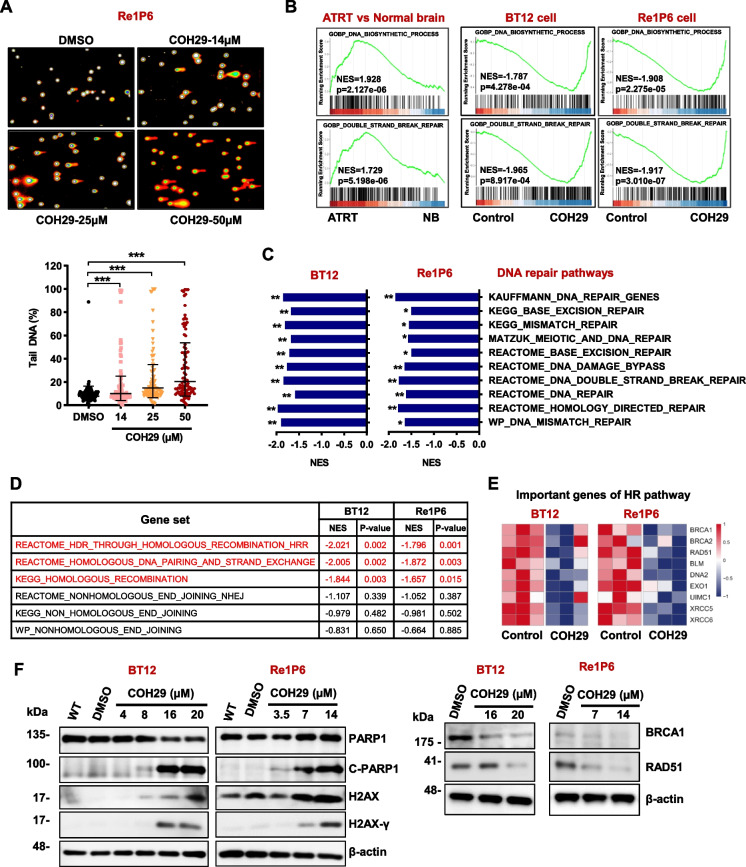
COH29 treatment activated DNA damage in ATRT. **A** Images and corresponding graph of comet assay after incubating Re1P6 cells with COH29. Comet assay data were analyzed using the Comet Score software. The pink and blue circles represent the DNA in the nucleus, while the orange represents the fragmented DNA. Representative of tail DNA (%) used DMSO treatment as control. Data are presented as the Geometric means with geometric SD of three independent experiments. ** *p* ≤ 0.01, *** *p* ≤ 0.001, Student’s t-test. **B** Gene set enrichment analysis of the DNA_biosynthetic_process and Double_strand_break_repair process in ATRT samples and BT12, Re1P6 cells after being treated with COH29. **C**, **D** GSEA analysis of the C2 Curated dataset indicated DNA repair pathways (**C**), or HR, NHEJ pathways (**D**) in COH29-treated cells in comparison with the controls. NES normalized enrichment score, p. adjusts value *p < 0.05, **p < 0.01. **E** Heatmap represents the expression of genes that play an important role in the HR pathway in BT12 and Re1P6 treated cells versus control. **F** Immunoblotting for DNA damage and HR markers of BT12 and Re1P6 cells treated with COH29 in 72 h and 48 h, respectively

It has been reported that COH29 induced the DNA damage in breast cancer cells [37]. To elucidate the effects of COH29 treatment, we next measured DNA strand breaks in ATRT cells with comet assay. Compared with DMSO control, COH29 significantly induced DNA damage, as evidenced by comet tails and the percentage of tail DNA (Fig. 6A and S6A). To further investigate the mechanisms under the anti-cancer effects of COH29 in ATRT, Re1P6 and BT12 cells were treated with COH29 at the indicated dose for 48 h, then the cell lysates were collected for RNA-seq. Based on the in silico analysis results, the effect of COH29 on the DNA replication and DNA repair system was found. Gene set enrichment analysis (GSEA) with ATRT patient’s database indicated that DNA biosynthetic process and double-strand break repair were activated (NES = 1.928 and 1.729, respectively). Impressively, these two processes were strongly suppressed in BT12 cell (NES = -1.787 and-1.965, respectively) and Re1P6 cell (NES = -1.908 and -1.917, respectively) after being treated with COH29 (Fig. 6B). Besides, the analyzed results of GSEAs C2 Curated gene sets revealed that the DNA repair signaling signatures were downregulated when ATRT cells were treated with COH29 (Fig. 6C). This finding indicated that COH29 treatment suppresses DNA repair system and induces DNA break in ATRT cell. Moreover, the two main mechanisms for repairing double strand breaks: Homologous recombination (HR) and classical nonhomologous end joining (NHEJ), particularly the HR signaling pathway, were highly activated in ATRT patients (Fig. S6B). However, both mechanisms were inhibited upon the COH29 treatment in ATRT cells, especially with a more dramatic suppression in the HR pathway (Fig. 6D). These results lead us to hypothesize that COH29 treatment not only causes DNA damage but also inhibits the HR pathway. Indeed, the expression of important genes in HR pathway including BRCA1, BRCA2, Rad51, BLM, DNA2, EXO1, UIMC1 (RAP80), XRCC5 (Ku80) and XRCC6 (Ku70) were downregulated in COH29-treated cells (Fig. 6E). These results were further confirmed by immunoblotting which revealed that the expression of DNA damage markers including cleaved PARP1 and H2AX-γ were increased with COH29 treatment (Fig. 6F and S6C). Contrarily, DNA repair markers BRCA1 and RAD51 were downregulated in COH29-treated cells (Fig. 6F and S6C). Altogether, our data suggest that COH29 treatment promotes DNA damage, inhibits DNA repair and induces apoptosis, demonstrating that COH29 has a strong anti-cancer effect on ATRT.

### Positive correlation between RRM2 and BRCA1 levels in ATRT

**Fig. 7:**
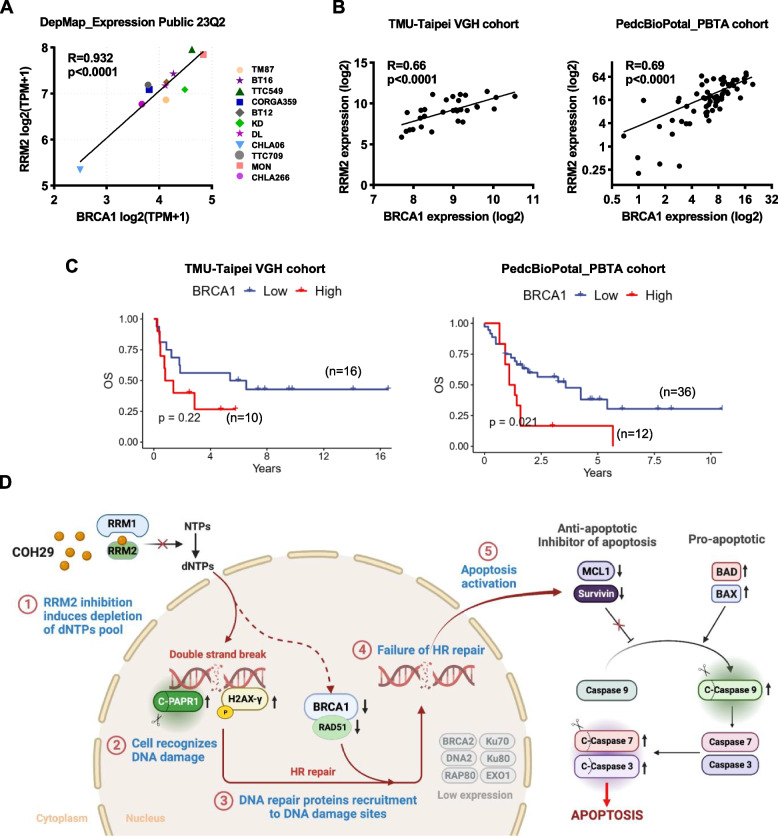
Proposed mechanisms of RRM2 inhibition by COH29 in ATRT cells via DNA damage induction and apoptosis activation. **A** The correlation of mRNA gene expression between RRM2 and BRCA1 in eleven ATRT cell lines. Data were analyzed from the project Expression Public 23Q2 dataset (DepMap). **B** Pearson correlation test between the RNA expression level of BRCA1 and RRM2 in human TMU-Taipei VGH cohort (*n* = 28) and PedcBioPortal_PBTA_cohort (*n* = 67). RNA expression was normalized using logarithm base 2. **C** The correlation of BRCA1 mRNA level with patient’s overall survival (OS) in TMU-Taipei VGH cohort (*n* = 26), and PBTA cohort from PedcBioPortal (*n* = 48). **D** Proposed schematic of COH29 treatment induces DNA damage and activates apoptosis in ATRT

BRCA1 plays a crucial role in repairing and recovering DNA damage induced by COH29 in breast cancer cells [37]. The findings that BRCA1 expression was downregulated by RRM2 inhibition in ATRT cells led us to further test the correlation between RRM2 and BRCA1 expression in ATRT cell lines. Analysis of DepMap_Expression Public 23Q2 revealed that BRCA1 expression was strongly associated with RRM2 expression in ATRT cell lines (*R* = 0.932, *p* < 0.0001) (Fig. 7A). In addition, a positive correlation was also found in the TMU-Taipei VGH cohort (*R* = 0.66, *p* < 0.0001) and PedcBioPortal_PBTA cohort (*R* = 0.69, *p* < 0.0001) (Fig. 7B). Furthermore, similar to the correlation between RRM2 expression level and patient overall survival, ATRT patients with high BRCA1 expression also had a shorter survival time than those with low expression (Fig. 7C). ATRT is characterized by the inactivation of SMARCB1, a crucial subunit of the SWI/SNF complex. Our bioinformatics analysis revealed a negative correlation between RNA expression of SMARCB1 and levels of both RRM2 and BRCA1 in the TMU-Taipei VGH cohort, PedcBioPortal_PBTA_cohort, and ATRT cell lines (Fig. S7). However, the precise mechanisms through which the SWI/SNF complex or its components regulate RRM2 or BRCA1 in ATRT remain unclear. Various explanations exist, but it has been proposed that the SWI/SNF complex interacts with chromatin, facilitating the binding of transcriptional machinery and thereby regulating gene expression [38]. This insight provides valuable cues and guides further investigation into the impact of SWI/SNF proteins on the expression of RRM2 and BRCA1. All these data note that there appears to be a linear relationship between RRM2 and BRCA1, which was further implicated the anti-cancer effects of COH29 via increasing DNA damage and decreasing the HR responses in ATRT.

Taken together, our study suggest that RRM2 may function as an oncogene and play vital role in ATRT survival and progression, making it a promising therapeutic target for this aggressive tumor.

## Discussion

ATRT is the most prevalent central nervous system tumor among infants under the age of one [5]. Despite the high incidence, there is currently no standardized treatment protocol for ATRT. The prognosis is generally very poor, with survival varying by age and treatment [4]. Therefore, there is an urgent need to discover an effective treatment for this cancer. Our studies on ATRT revealed the following key findings: (1) RRM2 exhibits elevated expression in ATRT compared with benign tumors or normal brain tissues, (2) increased RRM2 is correlated with poor survival rate among ATRT patients, (3) depletion of RRM2 inhibits ATRT cell growth, triggers DNA damage, and induces cell death, (4) the RRM2 inhibitor, COH29 suppresses tumor growth and prolongs survival in mice.

RRM2, a subunit of the RNR enzyme, is known to provide dNTPs for nuclear DNA replication and DNA repair [39]. Several studies have highlighted that RRM2 is overexpressed in various human malignancies such as prostate cancer [35], neuroblastoma [23], glioblastoma [14], breast cancer [40], etc. Moreover, upregulation of RRM2 has been associated with tumor angiogenesis, metastasis, and progression, as well as poor prognosis of patients [20–22]. Similar with these studies, our findings indicated that RRM2 was significantly upregulated in ATRT, and a higher level of RRM2 expression was positively correlated with poor patient survival. Additionally, the control of the dNTP pools primarily relies on RNR enzyme, and thus the RNR subunits are essential for supplying dNTP to replication fork and DNA damage sites. Imbalances in dNTP pools can lead to impaired DNA synthesis and faulty DNA repair [41, 42]. Numerous studies have highlighted that abnormal RRM2 degradation induces imbalance of the dNTP pool and instability of genome making RRM2 a potential target for cancer therapy [15, 20, 35]. Consistent with this, our study observed that RRM2 depletion by COH29 increases the percentage of DNA tails in the comet assay. In addition, we show dysregulation of two DNA damage markers, PARP1 and H2AXγ. Poly(ADP-ribose) polymerase 1 (PARP1 or ARTD1), was identified as one of the earliest proteins recruited to the DNA damage site. PARP1 has diverse roles in DNA damage response (DDR), including repair of single-strand breaks (SSBs) and double-strand breaks (DSBs), stabilization of DNA duplication, and chromatin remodeling [43]. Another key component of DDR, especially for DSBs is H2AX, a member of the histone H2A family. H2AX is recruited to DSB sites and rapidly phosphorylated to form H2AXγ. This phosphorylation event facilitates the recruitment and activation of other signals involved in DNA damage repair [44]. To further understand the underlying mechanisms, we employed bioinformatics analysis to explore the DNA damage-related signaling pathways. The GSEA analyzed results of DNA damage-related signaling profiling showed significant enrichment of DNA-repair pathways in ATRT patients. However, after treatment of ATRT cells with COH29, both the DNA repair pathways and DNA synthesis process were suppressed. These data suggest that RRM2 inhibition induces DSBs in ATRT cells, which leads to abnormal replication fork processing, chromosome breakage, or telomere de-protection.

In mammalian cells, there are two major mechanisms of DSB repair NHEJ and HR [45]. In our study, gene enrichment analysis revealed that the HR signaling pathway was significantly suppressed in COH29-treated cells compared with control groups. HR is a multistep process that begins with the activation of ATM, PARP1, and H2AX followed by the involvement of many other proteins [46]. The key facilitators of HR are breast cancer 1 (BRCA1) and RAD51. During HR, BRCA1 not only controls the initial step of DSB excision but also plays a role in loading RAD51 onto DNA damage site. RAD51 plays a central role in DNA filament formation and D-loop formation, in which DNA synthesis is initiated to replace the DNA surrounding the former break site [45, 47]. Consistent with other studies [48–51], our western blot combined with molecular profiling confirmed that RRM2 depletion reduced the levels of BRCA1 and RAD51 proteins, thereby suppressing HR in ATRT cells.

Mechanisms underlying the correlation between RRM2 and BRCA1 remains unclear. Studies in breast cancer have shown that COH29 induces greater DSBs and DNA-damage response in BRCA1-deficient cells than in normal cells. Furthermore, these studies reported that wild-type cells are less sensitive to COH29 treatment than BRCA1-deficient cells in vitro and in vivo [37]. Another study in glioblastoma showed that BRCA1 regulates RRM2 expression via E2F1, and RRM2 inhibition mimics the phenotype of BRCA1-loss in glioblastoma cells [15]. In our study, positive correlation between RRM2 and BRCA1 mRNA expression was observed, and ATRT patients with elevated levels of BRCA1 exhibited shorter survival times. However, further studies are needed to fully elucidate the relation between these two genes in ATRT. It is noteworthy that treatment with COH29 in ATRT cells resulted in decreased BRCA1 expression. This observation suggests that COH29 treatment not only causes DNA damage but also reduces the levels of key components of the DNA repair machinery. As a consequence, the efficiency of HR may be affected. These findings support the potential of COH29 as an effective anti-tumor drug in ATRT.

The fate of cells, whether it continuous to survive or undergoes programmed death such as apoptosis, necrosis, autophagy, and senescence, is determined by the process of DDR and the outcome of DNA repair. The most current paradigm states that if DNA repair fails, the cells die by activating one of the programmed death pathways, usually apoptosis. Therefore, numerous therapies targeting DDR have been investigated in the hope that it could destabilize the cancer genome and activate cell death process [52, 53]. RRM2, a vital player in DNA synthesis and repair, has emerged as a promising therapeutic target for cancer treatment [27]. Many studies have reported that the inhibition of RRM2 induces DDR and activates apoptosis [15, 35, 54]. In this study, we demonstrated that depletion of RRM2 can induce apoptosis in ATRT cells. The induction of apoptosis by RRM2 inhibition was further supported by in vivo experiments. COH29 significantly suppressed tumor growth and prolonged the survival of mice in the orthotropic ATRT mouse model. Similar to the results of ATRT cell, cleavage of Caspase 3 was significantly increased in tumor tissues of COH29-treated mice. Additionally, as mentioned earlier, PARP1 plays a crucial role in DDR, but its cleavage also serves as a marker for cells undergoing apoptosis. Previous studies have demonstrated that caspase 3 and 7, in their active forms, cleave PARP1 (110 kDa) between the amino acids Asp214 and Gly215, resulting in the separation of amino-terminal DNA-binding domain (24 kDa) from the carboxyterminal catalytic domain (89 kDa) [55, 56]. Once cleaved, PARP1 loses its ability to repair DNA damage, while the 89 kDa fragment containing most of its functional domains is translocated from the nucleus to the cytoplasm, thereby promoting apoptosis [57, 58]. Our Western blot analysis results showed a remarkable increase in cleaved PARP1 (89 kDa) in three ATRT cell lines treated with COH29. These findings strongly suggest a link between the downregulation of DNA damage repair and apoptosis in the presence of abnormally active RRM2. Collectively, our study demonstrates that RRM2 depletion leads to genomic instability, suppresses DDR, and induces apoptosis in ATRT cells (Fig. 7D).

There are two notable limitations in this study that could be addressed in future research. Firstly, our study only focused on elucidating the mechanisms underlying DNA damage and apoptosis in ATRT. However, our in vitro study also showed a significant effect on cell migration upon RRM2 inhibition. Although our animal models, visualized through MRI, effectively delineated tumor location and size in the brain, this imaging method falls short in assessing the metastatic status of mice. Therefore, future studies should delve into investigating tumor metastasis and the mechanisms governing cell migration under RRM2 inhibition in ATRT. Secondly, the intricate interplay among the DNA damage, cell cycle, and apoptosis pathways is evident. Depending on the nature and complexity of DNA damage, cell cycle checkpoints are activated, modulating gene expression through transcriptional or translational changes. Cells with irreparable DNA lesions may undergo permanent cell-cycle arrest or apoptosis [59]. Given RRM2’s pivotal role in dNTP synthesis and its influence on the cell cycle [60, 61], our study demonstrated that RRM2 inhibition induced DNA damage, leading to subsequent apoptosis in ATRT. However, the intricate connections between these two pathways and their relationship with the cell cycle remain elusive. The complexity of cell cycle mechanisms necessitates thorough and comprehensive investigations to discern the impact of RRM2 depletion on these three pathways.

## Conclusions

In summary, this study provides evidence for the overexpression and oncogenic role of RRM2 in ATRT. Its high expression levels are associated with poor survival outcomes in ATRT patients. Perturbation of RRM2 reserves its oncogenic activities, including cell proliferation, colony formation, and migration. In addition, the inhibition of RRM2 activates DNA damage, suppresses the homologous recombination pathway, and induces cell death. The observed sensitivity of ATRT cells to COH29 in vitro and in vivo supports the potential application of this drug in clinical trials as a novel treatment for ATRT.

### Supplementary Information

**Additional file 1: Figure S1.** Expression of candidate genes in ATRT and the essential of RRM2 for ATRT cell lines survival. **Figure S2.** Knockdown of RRM2 suppressed cell proliferation in the CHLA266 cell line. **Figure S3.** COH29 showed an anticancer effect on CHLA266 cells. **Figure S4.** Body weight of ATRT mice. **Figure S5.** COH29 treatment induced apoptosis in ATRT cells. **Figure S6.** COH29 treatment activated DNA damage in ATRT cells. **Figure S7.** The correlation of RNA expression between SMARCB1 and RRM2, BRCA1.

## Data Availability

The datasets used and/or analyzed during the current study are available from the corresponding author on reasonable request.

## Abbreviations

ATRT: Atypical teratoid rhabdoid tumors
RRM2: Ribonucleotide reductase regulatory subunit M2
CNS: Central nervous system
SMARCB1: SWI/SNF related, matrix associated, actin dependent regulator of chromatin, subfamily b, member 1
SMARCA4: SWI/SNF related, matrix associated, actin dependent regulator of chromatin, subfamily a, member 4
RNR: Ribonucleotide reductase
CHK1: Checkpoint kinase 1
PPI: Protein-protein interaction
TMUH: Taipei Medical University Hospital
VGH: Taipei Veterans General Hospital
shRNA: Short hairpin RNA
DMSO: Dimethyl sulfoxide
IC50: Half-maximal inhibitory concentration
MRI: Magnetic resonance imaging
PDX: Patient-derived xenograft
RNA-seq: RNA sequencing
SD: Standard deviation
FGFR1: Fibroblast growth factor receptor 1
TOP2A: DNA topoisomerase II alpha
KIF20A: Kinesin family member 20A
PBK: PDZ binding kinase
TTK: TTK protein kinase
TGFB3: Transforming growth factor beta 3
MUC1: Mucin 1, cell surface associated
RRM1: Ribonucleotide reductase catalytic subunit M1
RRM2B: Ribonucleotide reductase regulatory TP53 inducible subunit M2B
IHC: Immunohistochemistry
MCL1: MCL1 apoptosis regulator, BCL2 family member
BAX: BCL2 associated X, apoptosis regulator
BAD: BCL2 associated agonist of cell death
GSEA: Gene set enrichment analysis
HR: Homologous recombination
NHEJ: Nonhomologous end joining
BRCA1: BRCA1 DNA repair associated
BRCA2: BRCA2 DNA repair associated
Rad51: RAD51 recombinase
BLM: BLM RecQ like helicase
DNA2: DNA replication helicase/nuclease 2
EXO1: Exonuclease 1
UIMC1: Ubiquitin interaction motif containing 1
XRCC5: X-ray repair cross complementing 5
XRCC6: X-ray repair cross complementing 6
PARP1: Poly(ADP-ribose) polymerase 1
H2AX: H2A.X variant histone
dNTPs: Deoxynucleoside triphosphate
DDR: DNA damage response:
SSBs: Single-strand breaks
DBSs: Double-strand breaks
