# Supplementary material for: Targeting of RRM2 suppresses DNA damage response and activates apoptosis in atypical teratoid rhabdoid tumor
Source: J Exp Clin Cancer Res. 2023 Dec 20;42:346. doi: 10.1186/s13046-023-02911-x (PMC10731702; doi:10.1186/s13046-023-02911-x)
Supplement: Supplementary file 1 — Additional file 1: Figure S1. Expression of candidate genes in ATRT and the essential of RRM2 for ATRT cell lines survival. Figure S2. Knockdown of RRM2 suppressed cell proliferation in the CHLA266 cell line. Figure S3. COH29 showed an anticancer effect on CHLA266 cells. Figure S4. Body weight of ATRT mice. Figure S5. COH29 treatment induced apoptosis in ATRT cells. Figure S6. COH29 treatment activated DNA damage in ATRT cells. Figure S7. The correlation of RNA expression between SMARCB1 and RRM2, BRCA1. [file 13046_2023_2911_MOESM1_ESM.docx]

**Targeting of RRM2 suppresses DNA damage response and activates apoptosis in atypical teratoid rhabdoid tumor**

Le Hien Giang, Kuo-Sheng Wu, Wei-Chung Lee, Shing-Shung Chu, Anh Duy Do, Chun A. Changou, Huy Minh Tran, Tsung-Han Hsieh, Hsin-Hung Chen, Chia-Ling Hsieh, Shian-Ying Sung, Alice L. Yu, Yun Yen, Tai-Tong Wong, and Che-Chang Chang*

* Corresponding authors:

To whom correspondence should be addressed. Contact should be made with:

Dr. Che-Chang Chang, The Ph.D. Program for Translational Medicine, College of Medical Science and Technology, Taipei Medical University, 6F., Education & Research Building, Shuang-Ho Campus, No. 301, Yuantong Rd., Zhonghe Dist., New Taipei City, 23564, Taiwan.

E-mail: ccchang168@tmu.edu.tw; Tel: 886-2-66202589 ext.10602

This additional file including supplementary figures and figure legends

Figure S1. Expression of candidate genes in ATRT and the essential of RRM2 for ATRT cell lines survival.

Figure S2. Knockdown of RRM2 suppressed cell proliferation in the CHLA266 cell line.

Figure S3. COH29 showed an anticancer effect on CHLA266 cells.

Figure S4. Body weight of ATRT mice.

Figure S5. COH29 treatment induced apoptosis in ATRT cells

Figure S6. COH29 treatment activated DNA damage in ATRT cells

Figure S7. The correlation of RNA expression between SMARCB1 and RRM2, BRCA1

**Supplementar
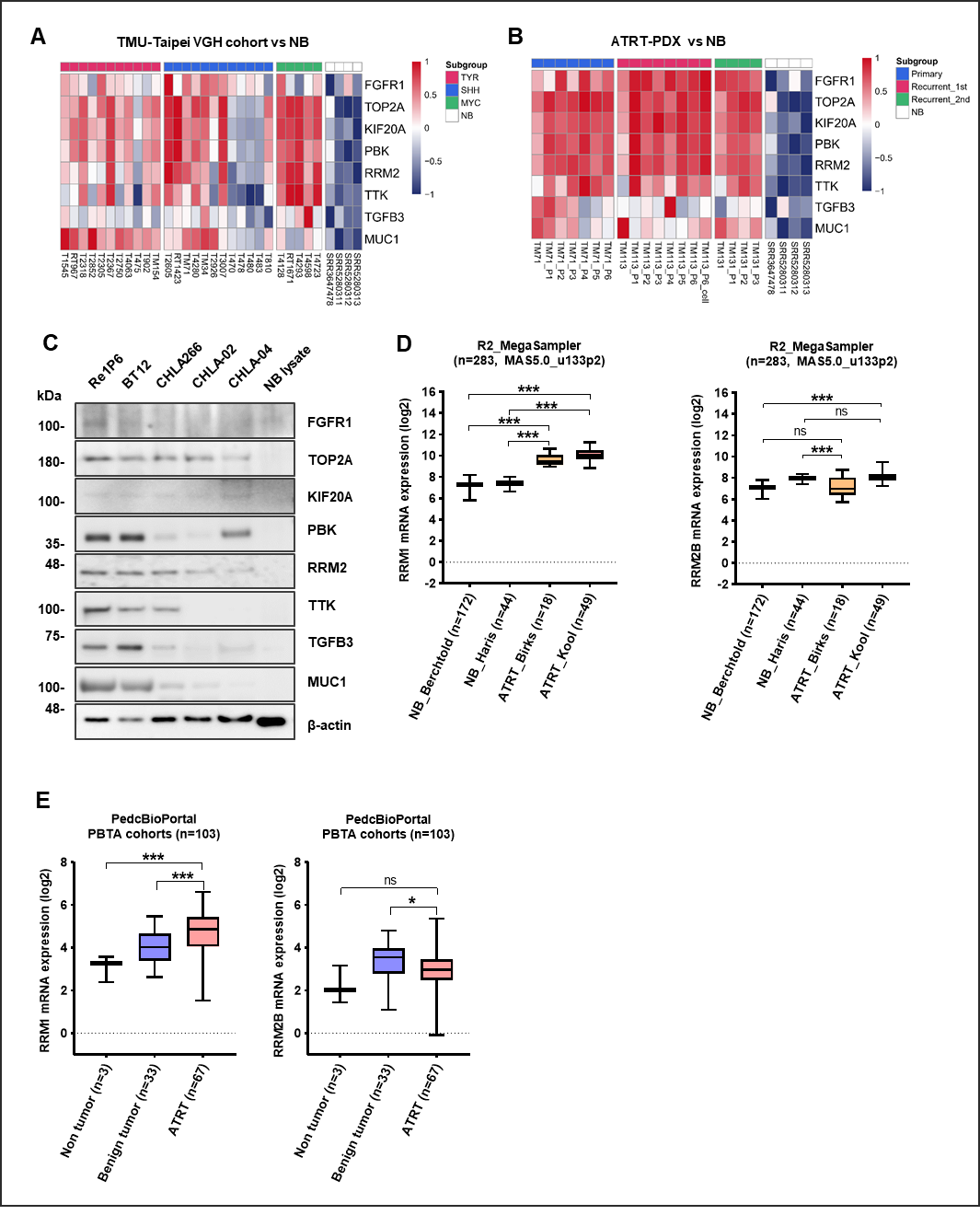
y Figure 1**

**Figure S1. Expression of candidate genes in ATRT and the essential role of RRM2 for ATRT cell lines survival.** **(A, B)** Subgroup expression profile of eight target genes in TMU-Taipei VGH cohort (n=28) (A), and ATRT PDX samples (primary, 1^st^ recurrent, and 2^nd^ recurrent) (B) compared with normal brain tissues. **(C)** Protein expression of eight target genes in five ATRT cell lines and human normal brain tissue lysate (NB lysate). **(D)** Expression levels of RRM1 and RRM2B mRNA in human ATRT and normal brain tissues in public data set using R2 Platform. **(E)** Expression levels of RRM1 and RRM2B mRNA in human ATRT, normal brain, and benign tumor tissues from PedcBioPortal data set. Bar indicates the mean mRNA levels of each group, data are presented as min to max, ns non-significant, **p* < 0.05, ***p* < 0.01, ****p* < 0.001, Tukey's multiple comparisons test.

**Supplementary Figure 2**


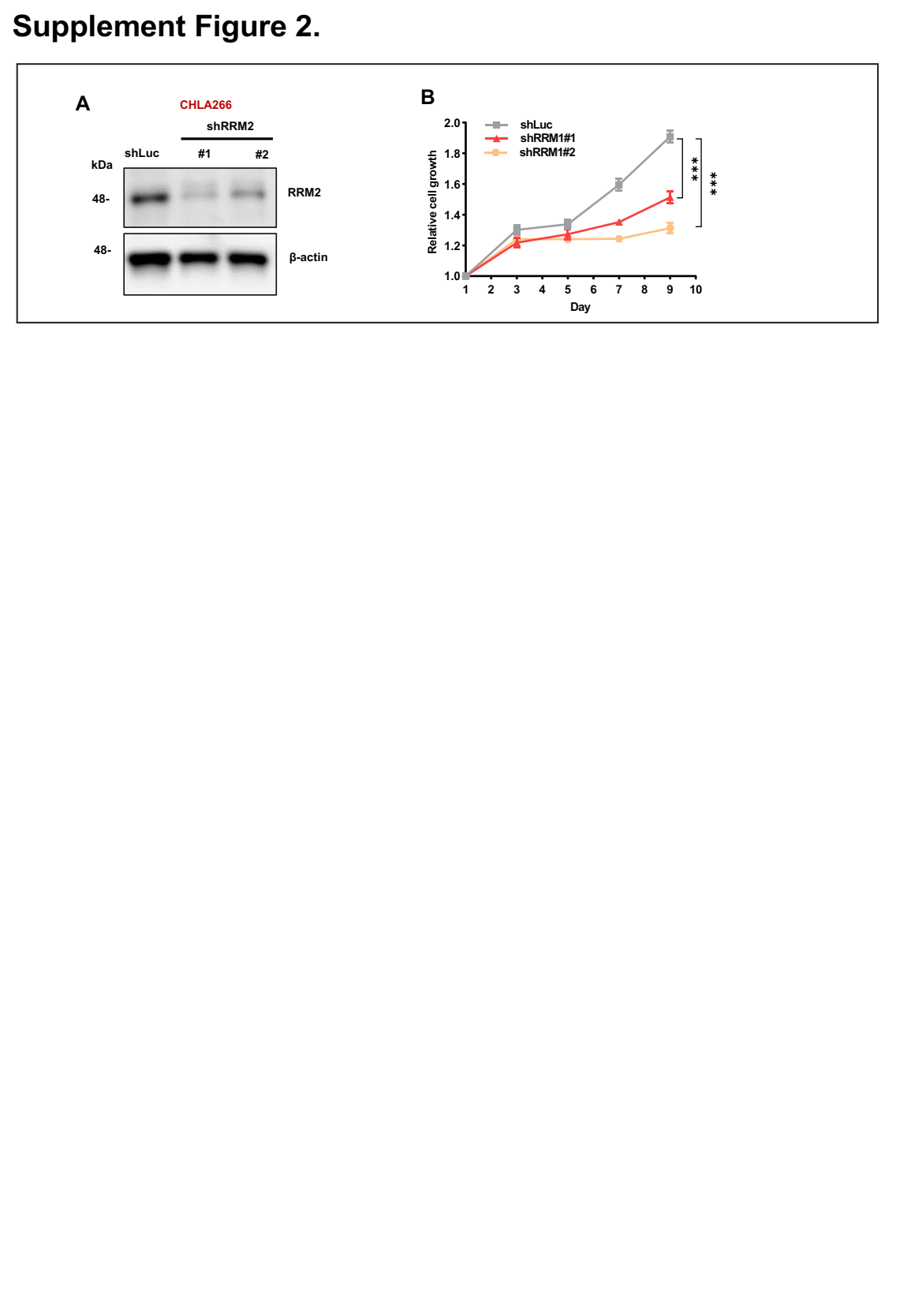


**Figure S2. Knockdown of RRM2 suppressed cell proliferation in the CHLA266 cell line. (A)** Immunoblotting analyzed RRM2 knockdown efficiency. **(B)** CHLA266 cell proliferation after knockdown RRM2 by shRRM1#1 and shRRM2#2, shLuc was used as control Data are presented as the mean ± standard deviation of triplicated independent experiments, ****p* ≤ 0.001, Student's t-test.

**Supplementary Figure 3**

**Figure S3. COH29 showed an anticancer effect on CHLA266 cells.** **(A, B)** Proliferation assay (A) and colony formation assay (B) of CHLA266 cell after being treated with COH29. **(C)** Wound healing assay after CHLA266 cell was treated with COH29. The relative would closure was quantified by ImageJ. **(D)** Transwell assay analyzed the migration ability of CHLA266 cells after being treated with COH29 in 7 and 14 μM. A relative rate of cell migration was analyzed from the number of the cells migrating per chamber compare with the control. In all experiments, 0.1% DMSO was used as the control. Data are presented as the SD deviation of triplicated independent experiments, ns non-significant, **p* ≤ 0.05, ***p* ≤ 0.01, ****p* ≤ 0.001, Student's t-test.

**Supplementary Figure 4**

**
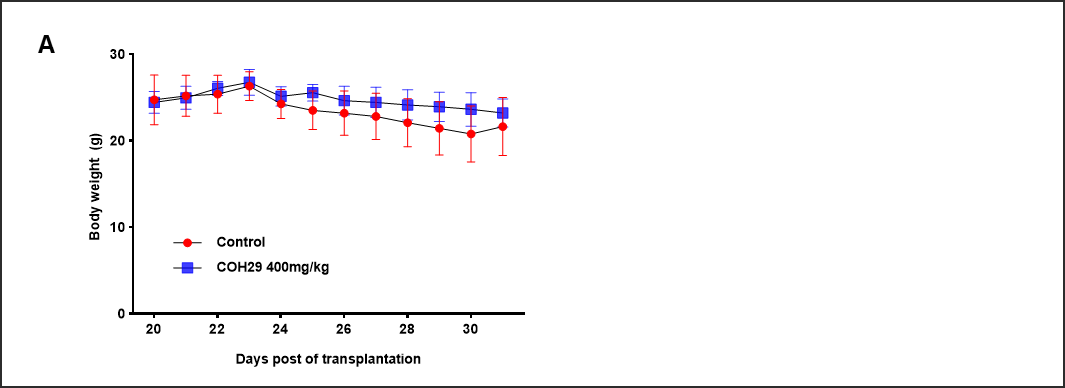
**

**Figure S4. Body weight of ATRT mice. (A)** Monitoring of mice body weight in orthotopic ATRT model. Error bar, SD.

**Supplementary Figure 5**


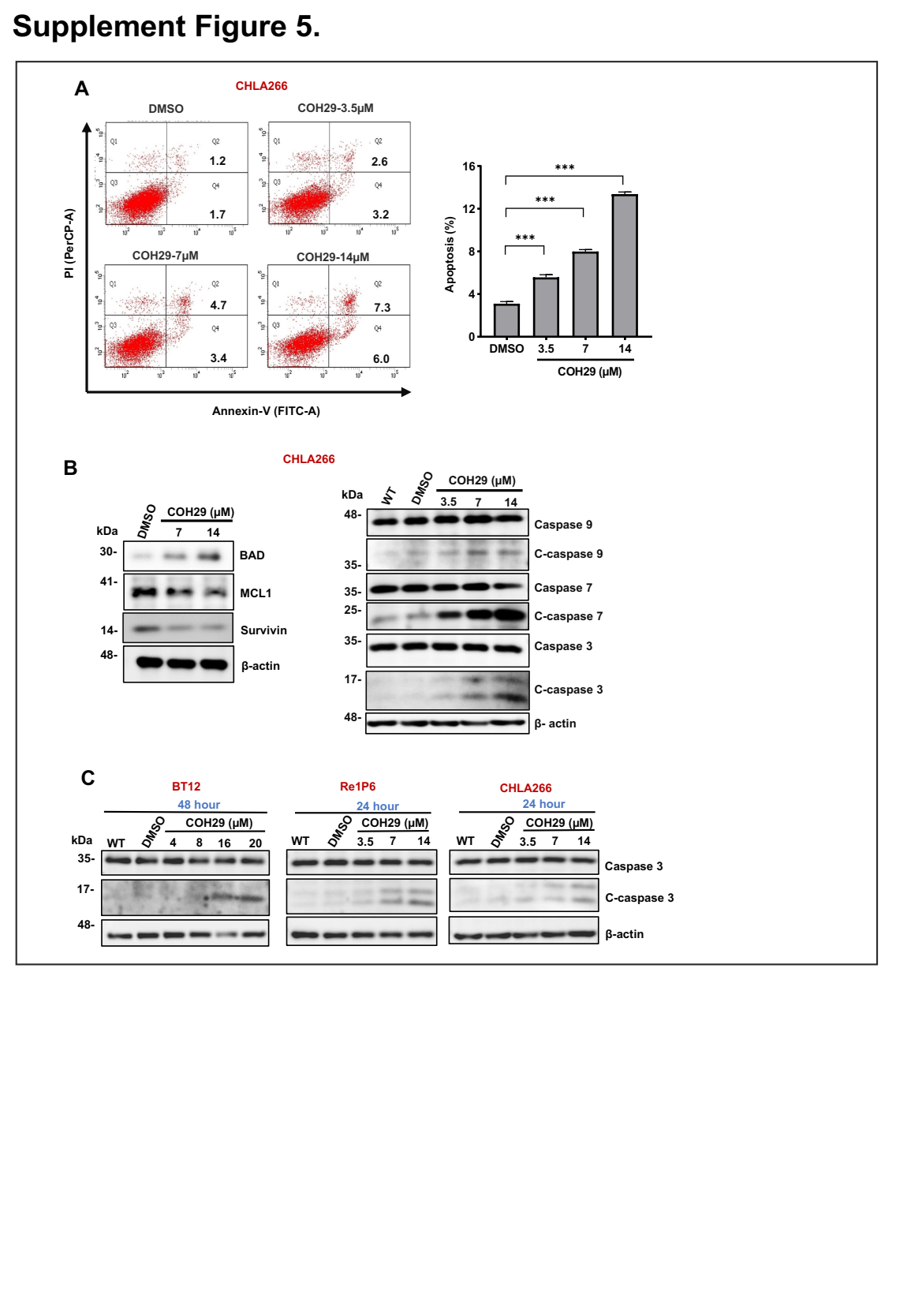


**Figure S5**. **COH29 treatment induced apoptosis in ATRT cells.** **(A)** Flow cytometry analyzed the percentage of CHLA 266 apoptotic cells after treatment with COH29. Cells were treated with COH29 for 48 h, and 0.1% DMSO was used as a control. Data are presented as mean ± SD of three independent experiments. ****p* ≤ 0.001, Student’s t-test. (**B**) Immunoblotting for apoptosis markers of CHLA266 cells treated with COH29 in 48h. **(C)** Immunoblotting for cleaved-caspase 3 after 48h (for BT12) or 24h (for Re1P6 and CHLA266) treated with COH29.

**Supplementary Figure 6**


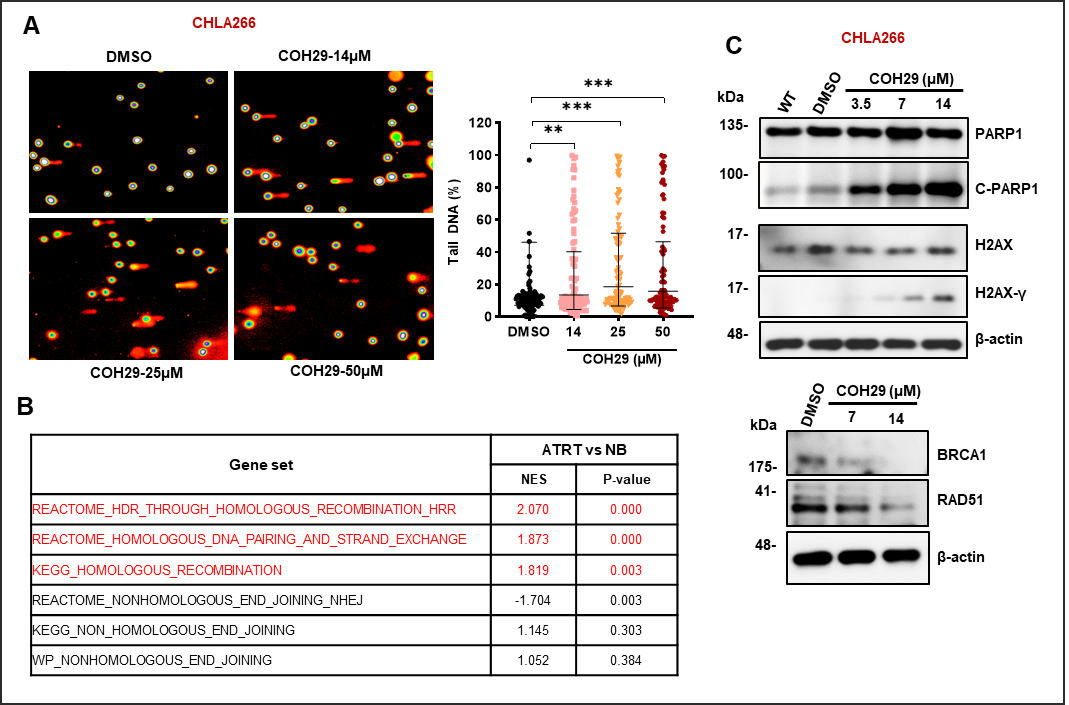


**Figure S6**. **COH29 treatment activated DNA damage in ATRT cells**. **(A)** Comet assay result after incubating CHLA266 cells with COH29. Comet assay data were analyzed using the Comet Score software. The pink and blue circles represent the DNA in the nucleus, while the orange represent the fragmented DNA. Representative of tail DNA (%) used DMSO treatment as control. Data are presented as the Geometric means with geometric SD of three independent experiments. ** *p* ≤ 0.01, *** *p* ≤ 0.001, Student’s t-test. **(B)** GSEAs of the C2 Curated dataset revealing the downregulation of gene signatures representing HR and NHEJ gene sets in ATRT in comparison with normal brain. NES normalized enrichment score, p. adjusts value **p* < 0.05, ***p* < 0.01. **(C)** Immunoblotting for DNA damage markers of CHLA266 cells treated with COH29 in 48h.

**Supplementary Figure 7**

**
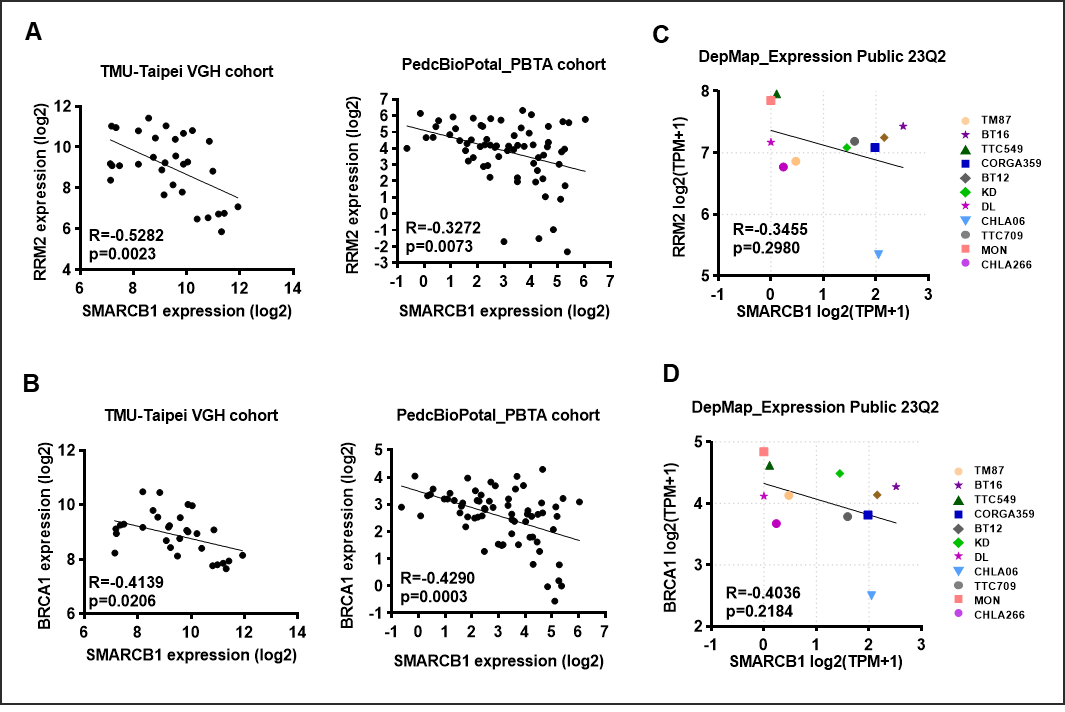
**

**Figure S7**. **The correlation of RNA expression between SMARCB1 and RRM2, BRCA1.** **(A, B)** Pearson correlation test between the RNA expression level of SMARCB1 and RRM2 (A), SMARCB1 and BRCA1 (B) in human TMU-Taipei VGH cohort (n=28) and PedcBioPortal_PBTA_cohort (n=67). RNA expression was normalized using logarithm base 2. **(C, D)** The correlation of mRNA gene expression between SMARCB1 and RRM2 (C), SMARCB1 and BRCA1 (D) in eleven ATRT cell lines. Data were analyzed from the project Expression Public 23Q2 dataset ([DepMap](https://depmap.org/)).
